# Protocol for a mixed methods study investigating the impact of investment in housing, regeneration and neighbourhood renewal on the health and wellbeing of residents: the GoWell programme

**DOI:** 10.1186/1471-2288-10-41

**Published:** 2010-05-11

**Authors:** Matt Egan, Ade Kearns, Phil Mason, Carol Tannahill, Lyndal Bond, Jennie Coyle, Sheila Beck, Fiona Crawford, Phil Hanlon, Louise Lawson, Jennifer McLean, Mark Petticrew, Elena Sautkina, Hilary Thomson, David Walsh

**Affiliations:** 1 Medical Research Council/Chief Scientist Office Social & Public Health Sciences Unit, 4 Lilybank Gardens, Glasgow, UK; 2 Department of Urban Studies, University of Glasgow, 25 Bute Gardens, Glasgow, UK; 3 Glasgow Centre for Population Health, 1st Floor, House 6, 94 Elmbank Street, Glasgow, UK; 4 NHS Health Scotland, Elphinstone House, 65 West Regent Street, Glasgow, UK; 5 Public Health and Health Policy, 1 Lilybank Gardens, University of Glasgow, Glasgow, UK; 6 Public and Environmental Health Research Unit, London School of Hygiene & Tropical Medicine, Keppel St., London, UK

## Abstract

**Background:**

There is little robust evidence to test the policy assumption that housing-led area regeneration strategies will contribute to health improvement and reduce social inequalities in health. The GoWell Programme has been designed to measure effects on health and wellbeing of multi-faceted regeneration interventions on residents of disadvantaged neighbourhoods in the city of Glasgow, Scotland.

**Methods/Design:**

This mixed methods study focused (initially) on 14 disadvantaged neighbourhoods experiencing regeneration. These were grouped by intervention into 5 categories for comparison. GoWell includes a pre-intervention householder survey (n = 6008) and three follow-up repeat-cross sectional surveys held at two or three year intervals (the main focus of this protocol) conducted alongside a nested longitudinal study of residents from 6 of those areas. Self-reported responses from face-to-face questionnaires are analysed along with various routinely produced ecological data and documentary sources to build a picture of the changes taking place, their cost and impacts on residents and communities. Qualitative methods include interviews and focus groups of residents, housing managers and other stakeholders exploring issues such as the neighbourhood context, potential pathways from regeneration to health, community engagement and empowerment.

**Discussion:**

Urban regeneration programmes are 'natural experiments.' They are complex interventions that may impact upon social determinants of population health and wellbeing. Measuring the effects of such interventions is notoriously challenging. GoWell compares the health and wellbeing effects of different approaches to regeneration, generates theory on pathways from regeneration to health and explores the attitudes and responses of residents and other stakeholders to neighbourhood change.

## Background

### Regeneration and health

The World Health Organisation (WHO) states that housing characteristics, including the interlinked dimensions of household, dwelling, community and neighbourhood environment, have the capacity to affect individual health status through physical, mental or social mechanisms [1]. As poor health is associated with poorer living circumstances, there is a policy expectation that area regeneration and housing improvement strategies will contribute to health improvement and reduced social inequalities in health [2-4]. We have designed a study to test this hypothesis.

### Evidence from systematic reviews

There is limited evidence on the health impacts of housing improvement and regeneration. For example, a systematic review of the health impacts of housing improvement has found that improvements in respiratory, general and mental health have been observed following home warmth improvement measures, but these health improvements varied across studies [5]. Some of the reviewed studies reported that housing improvement was associated with positive impacts on socioeconomic determinants of health. An earlier systematic review found some evidence that housing improvements led to rent increases - a possible mechanism for adverse outcomes to low budget households with inadequate welfare protection [6]. Evidence on the health impacts of housing interventions that included neighbourhood improvements was found to be inconsistent or unclear.

Despite several systematic reviews, we are aware of little or no robust evidence on the positive and negative health effects of broader, multi-component housing-led area based regeneration. Other 'evidence gaps' include long term effects of housing improvement and area regeneration, the social patterning of effects (i.e. effects on health inequalities), the comparative effects of rehousing and housing improvement, and the mechanisms by which different interventions or combinations of interventions might lead to positive health outcomes [5,7,8].

### GoWell

The Glasgow Community Health and Wellbeing (GoWell) Research and Learning Programme has been designed to provide such evidence. GoWell focuses on a large, multi-faceted programme of housing investment and area regeneration across the city of Glasgow (Scotland). These interventions can be described as 'natural experiments.' At an early stage in the planning process, regeneration planners identified high level goals that included improvements in residents' health and health behaviours, and reduced health inequalities. This contributed to the development of GoWell (GoWell is the name of the study, not the regeneration programme).

GoWell is a research and learning programme that aims to investigate the impact of investment in housing, regeneration and neighbourhood renewal on the health and wellbeing of individuals, families and communities over a ten-year period. The Programme aims to establish the nature and extent of these impacts and the processes that have brought them about, to learn about the relative effectiveness of different approaches, and to inform policy and practice. It is a multi-component study with a comparative design. Although focused on regeneration in Glasgow, we aim to produce findings that are transferable to other regeneration settings.

This paper summarises GoWell's methods, focusing particularly on its *Community Health and Wellbeing Survey*, and discusses some of its strengths and limitations.

## Methods/Design

### Summary

GoWell may be described as an observational study of a complex intervention. It is a mixed methods study that includes both quantitative and qualitative methods. Comparison areas (all relatively deprived) are incorporated into GoWell's design so we can compare the effects of different approaches to regeneration in different areas of the city. These regeneration activities are planned and delivered by a variety of stakeholders rather than by the researchers.

GoWell has been designed to regularly produce findings on short term impacts to inform ongoing planning and delivery of the interventions being evaluated (i.e. formative evaluation), and more broadly transferable evidence of effects including short, medium and long term outcomes. (i.e. summative evaluation) [9].

### Setting

Glasgow is the largest city in Scotland and contains high concentrations of poverty, disadvantage and ill health [10,11]. Area based health inequalities are stark: for example life expectancy in the most disadvantaged areas of Glasgow has been estimated to be at least 15 years shorter than in the least disadvantaged areas [12-14].

Glasgow's urban landscape includes features familiar to many European cities. Many of its residents live in flats rather than houses, and most have either no garden or share a garden. Even some of the more popular inner-city gardened estates contain buildings that look like large semi-detached houses but actually contain 4 single-entry flats with shared gardens (called 'four-in-a-block' flats). Glasgow's socially disadvantaged areas include large post-war peripheral estates (made up of 'low-rise' tenement flats, houses and, to a lesser extent, 'high-rise' multi-storey flats); inner-city mass housing estates (mostly post-war multi-storey flats with some tenement flats); inner-city gardened estates (houses and 'four-in-a-blocks' mostly dating from the 1930s); and old neighbourhoods dominated by 19th and early 20th century tenement flats. There are also affluent neighbourhoods in the suburbs and inner-city with housing types that include houses, tenements and recently built multi-storey developments.

### Interventions

In 2003, over 80,000 socially rented homes in the city transferred from public ownership to a newly created not-for-profit organisation called Glasgow Housing Association (GHA), following a tenants' referendum. GHA became the largest provider of social housing in the city alongside a number of smaller providers (collectively known as 'Registered Social Landlords' or RSLs). The stock transfer paved the way for a city-wide regeneration investment programme spearheaded by GHA but involving other RSLs and partner organisations from other sectors.

Key elements of the programme include:

• **Housing improvement**: includes internal and external refurbishment based on surveyor's assessments of each property. Social rented homes receive the bulk of this investment, which is driven by a government requirement that all social housing meets the Scottish Housing Quality Standard by 2015 [15].

• **New builds**: building new homes, including socially rented and private sector homes in green and brown field sites. Plans include more owner occupied homes in areas dominated in the past by socially rented property (referred to as tenure diversification), especially in regeneration areas.

• **Transformational regeneration**: a neighbourhood-wide approach to regeneration planning (rather than improvements planned on a home-by-home basis) involving several or all of the following: relocation of residents, demolitions, new builds, housing improvement, tenure diversification, improvements to the physical neighbourhood environment, new/improved amenities and services, and community interventions. Typically, a Transformational Regeneration Area (TRA) would initially comprise between 1000-2000 households.

• **Local regeneration**: similar to transformational regeneration but targeting smaller pockets of disadvantage situated in larger neighbourhoods.

• **Community interventions (sometimes called 'wider actions')**: include employment and training initiatives, activities for young people, improved play areas, support for vulnerable people, addiction support, parent and child groups, financial advice and debt management, services for elderly residents, community buildings and resources, and other investments intended to strengthen and support communities in specific localities or across the city.

• **Community engagement and empowerment**: improving the way tenants are informed and consulted regarding decisions affecting their homes, neighbourhoods, communities and public services. Includes provision of information, surveys, consultation exercises and changes in housing management processes (including the gradual splitting of GHA into smaller local housing organisations working towards becoming independent Registered Social Landlords).

• **Wider effects**: it is hoped that transforming highly disadvantaged neighbourhoods and reducing social problems in those areas will benefit adjoining neighbourhoods.

### Scale and Cost of Intervention

Around three quarters of Glasgow's social housing needed improvement to meet the Scottish Housing Quality Target, including most of the 80,000 homes transferred to GHA, and homes managed by other Registered Social Landlords. Approximately 20,000 homes are to be demolished over a 15-year period following stock transfer (to 2018). GHA is to build approximately 3,000 new homes with a further 3,000 being provided by other RSLs within the city [16]. Private sector new builds are also planned but subject to market conditions.

The cost of these interventions in each area will be identified and tracked over time. Figures from GHA's 30 year business plan state that some £1,714 million of public money was invested in the initial housing stock transfer [17]. Of that, £900 million was historic debt which was written off. That left GHA with access to £814 million of public money and to £725 million of private borrowing (a bank loan to be repaid by 2033). Of the £814 million, £114 million had been allocated for demolitions, £113 million for new homes, £21 million for central heating and £100 million for capital works for owner occupiers. The balance, £466 million, along with annual rental income of some £200 million, is committed to home and neighbourhood improvements, providing housing services, community regeneration and running the Local Housing Association network and support services [17].

### Study Design

GoWell consists of a number of inter-connecting components (i.e. a community health and wellbeing survey; a longitudinal component; an ecological component; a qualitative study of governance, empowerment and participation; and nested evaluations of 'wider actions'). Detailed methodological descriptions of each component (some of which are themselves multi-method) would be too voluminous for a single paper. This paper will focus on GoWell's Community Health and Wellbeing Survey, which provides formative and summative data on the outputs, outcomes and health effects of different regeneration interventions at a neighbourhood level. Other components are detailed in Additional file 1 and will be described more fully in future papers.

The Community Health and Wellbeing Surveys are repeat cross-sectional surveys of (at baseline) approximately 6000 householders living in the 14 study areas using structured questionnaires. After each survey wave, the GoWell team select key findings/issues to explore further using qualitative research methods (12 focus groups after each wave). This selection can involve stakeholder engagement (e.g. through GoWell events, conferences, meetings, etc) to identify findings of particular interest to those delivering or receiving the interventions.

### Area selection and sample targets

**Figure 1:**
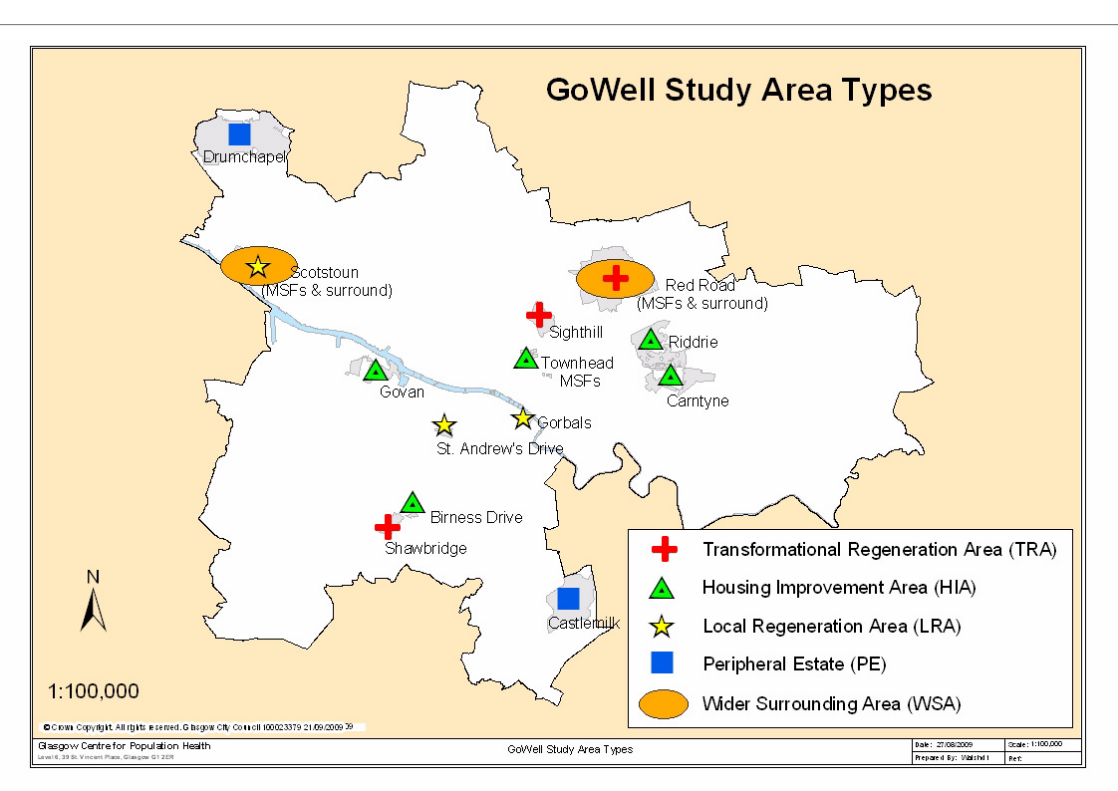
**Map of GoWell study areas**. Map of Glasgow showing the GoWell study areas (^© ^Crown Copyright. All rights reserved. Glasgow City Council, 100023379, 2009):

Fourteen areas were selected from lists of Glasgow neighbourhoods scheduled to receive one of five types of intervention package, with the key inclusion criteria being that intervention delivery would commence after the baseline survey (i.e. after September, 2006) [18]. Table 1 describes the intervention types and study areas. Table 2 summarises data on the number of households and tenure mix in each intervention area type at the time of the baseline survey. It shows that the Transformational and Local Regeneration Areas (TRAs and LRAs) were dominated by social rented dwellings; Housing Improvement Areas (HIAs) and Wider Surrounding Areas (WSAs) contained a broadly even split of social rented and private homes; whilst just over three quarter of the Peripheral Estates' (PEs) dwellings were social rented. Figure 1 is a map showing the location of study areas across the city.

**Table 1:** GoWell Intervention Types and Study Areas.

Name of Intervention area type	Intervention Types	No. of Areas	Description of Study Areas
Transformational Regeneration Areas (TRAs)	Transformational regeneration. Community interventions (wider actions). Engagement/empowerment.	3	3 inner-city mass housing estates
			
Local Regeneration Areas (LRAs)	Local regeneration. Community interventions (wider actions). Engagement/empowerment.	3	3 inner-city mass housing estates
			
Wider Surrounding Areas (WSAs)	Wider effects and housing improvement. Community interventions (wider actions). Engagement/empowerment.	2	2 inner-city gardened estates (surrounding a TRA and a LRA).
			
Housing Improvement Areas (HIAs)	Housing improvement. Community interventions (wider actions). Engagement/empowerment.	4*	2 inner-city gardened estates 2 inner-city mass housing estates
			
Peripheral Estates (PEs)	New builds (mostly private sector) Housing improvement. Community interventions (wider actions). Engagement/empowerment.	2	2 peripheral estates

* Note: in 2008, a fifth Housing Improvement Area (Birness Drive) was selected to collect data on more popular multi-storey flats - bringing the total number of GoWell areas to 15 for the second survey wave. However, only the original 14 areas can be compared with baseline findings.

**Table 2:** Number and percentage of social rented, private rented and owner occupied homes in GoWell intervention area types.

Area types	No. of social rented households (%)	No. of private rented households (%)	No. of owner occupied households (%)	Total
TRAs	4927 (96.3)	27 (0.5)	160 (3.1)	5114
LRAs	1719 (91.6)	35 (1.9)	122 (6.5)	1876
WSAs	2840 (45.6)	205 (3.3)	3185 (51.1)	6230
HIAs	2973 (53.8)	169 (3.1)	2381 (43.1)	5523
PEs	5345 (77.8)	93 (1.4)	1436 (20.9)	6874
Total	17804 (69.5)	529 (2.1)	7284 (28.4)	25617

Source: Glasgow City Council, Council Tax Register, August 2006.

### Power calculations

Power calculations for two-tail tests were carried out based on previous housing research [19,20]. We were interested in a range of outcomes with a range of prevalences. For different types of analysis we could differentiate between, or combine, various area types or subgroups within our study. To give an idea of the sorts of outcomes we could detect, the following examples were calculated. For a common condition (25% prevalence - for example, some common psychological symptoms), to detect a small reduction in prevalence to 20% with 80% power would require a sample of 2266 at a minimum. For a condition with a lower prevalence of 15% (e.g. some respiratory conditions), having 80% power to detect a reduction of 5% would require a sample of 1450. Changes may be more pronounced for some of GoWell's residential outcomes: a reduction from 25% to 15% would require a sample size of 540.

### Sampling

The 14 areas varied greatly in population size at baseline and it was expected that some would experience major population shifts during the study period due to demolition and new build programmes. Applying a single sampling target or sampling fraction for all the areas would have led to underpowered samples for some area types or over-sampling elsewhere. We set pragmatic sample targets for achieved responses (we initially assumed a 60% response rate) in each area and area type at every wave. The sample targets for the baseline survey were as follows: TRAs = 1750; LRAs = 750; WSAs = 700; HIAs = 1400; PEs = 1400. The TRAs and LRAs targets were particularly high (relative to the number of households in these areas) to maximize numbers for GoWell's longitudinal study component (see Additional file 1). Addresses were selected at random from the Royal Mail postal address file, or for smaller areas all residential addresses were selected. We sampled one householder per household (by most recent birthday, as required).

Most focus group participants have been (and will continue to be) participants of the cross-sectional survey (or in a small number of cases, their children). Sampling for the focus groups was generally purposive and dependent on the themes selected. Usually, fieldworkers aimed to recruit around 8 participants per focus group, with a target of around a dozen focus groups at each wave.

### Recruitment

Households selected for cross-sectional surveys were posted information sheets and letters inviting householders to take part. Fieldworkers then made at least 5 attempts (if necessary) to contact selected homes in person to seek consent to participate. A face-to-face questionnaire lasting around 40 minutes was verbally administered by fieldworkers at participants' homes with responses recorded using paper or CAPI (Computer Assisted Personal Interviewing).

Interpreters were available or assistance from other household members obtained if selected householders did not speak English (some contracted fieldworkers were also fluent in several languages common amongst UK non-English speakers). GoWell information leaflets were produced in English, Arabic, Urdu, Cantonese and Turkish.

Participants selected for post-survey focus groups were re-contacted, provided with information about the nature of the focus group and given the option of attending a session - held at local venues such as community halls or schools.

An incentive (e.g. £20 supermarket voucher) was provided for those attending focus groups/qualitative interviews. No financial incentive was offered to participants of the 2006 repeat cross-sectional survey, but a prize draw has been used for subsequent repeat cross-sectional and longitudinal surveys.

### Follow-up

The Community Health and Wellbeing component includes 4 survey waves (one baseline, 3 follow-up over a ten year period). The baseline survey was conducted in 2006 and wave 2 took place in 2008. Wave 3 is planned for 2011 and wave 4 for a period 2-3 years thereafter, to be agreed in light of pace of intervention implementation. The 2006 survey took place from April to July, with subsequent cross-sectional surveys also conducted in the summer months. Focus groups are scheduled for the Autumn months following the surveys.

### Outcome measures

When designing the study we considered the physical and mental health scores measured by the Short Form 12 version 2 questionnaire (SF-12) to be GoWell's primary outcome measures [21]. There are sound methodological reasons for having a primary outcome measure but in reality GoWell's stakeholders are interested in a wide range of outcomes, and their priorities change over time. As our understanding of the mechanisms and pathways from regeneration to health develops over the course of the study, so our range of outcomes has become more broadly defined.

**Figure 2:**
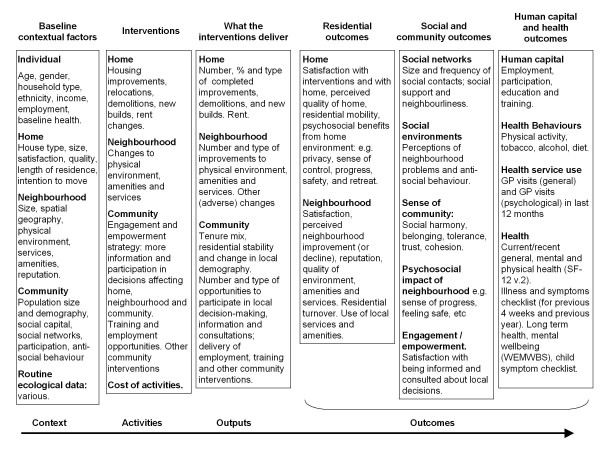
**Summary of GoWell outcomes**.

Figure 2 summarises categories of data collected by the Community Health and Wellbeing Survey. It includes self-reported items on neighbourhood contextual factors, interventions, outputs and outcomes, including health outcomes but also social and residential outcomes. We also collect relevant routine data to add to our understanding of these dimensions over the course of the study (see Additional file 1).

Details of the baseline questionnaire are provided in Additional file 2. The questionnaire contained mostly closed response questions (and a small number of open response questions). It is based on a questionnaire piloted in a previous study (SHARP [22]), which was itself adapted from other questionnaires. Some additional questions were added following consultations with stakeholders. Further details on the development of the questionnaire are available [23].

The questionnaire included items about respondents' households, homes, neighbourhoods, communities and local housing management. There were questions on residential outcomes, social and community outcomes, human capital, health behaviours, health service use, physical and psychological health during the previous 4 weeks and the previous 12 months. Most of these questions asked about the respondent's own health although parents were also asked about the health of their children. Subsequent cross-sectional and longitudinal GoWell questionnaires replicate most of the items in the baseline questionnaire to enable comparability [24]. The Warwick-Edinburgh Mental Wellbeing Scale (*WEMWBS*) was unavailable at baseline but was included from Wave 2 onwards to add a positive mental wellbeing measure to the more 'clinical' SF-12 mental health measures [21,25].

### Data Preparation

After each survey, data are range and quality control-checked and back-checked in a process that includes one-in-five respondents being contacted by telephone to verify the manner of conduct of the interview and to double-check a selected number of responses for accuracy. All 'errors' found in the back-checking exercise are corrected. Data cleaning continues throughout the analysis.

### Weighting

To ensure as far as possible that our sample is representative of key features of the population, we developed a set of weights (numerical coefficients) for all of the cases by which the responses of people who possessed characteristics that were under-represented in our sample were given greater importance, while the importance of responses from residents with over-represented characteristics was downplayed. The choice of weights took into account the availability of data upon which weighting could reliably be based and five were decided upon as being most relevant to findings related to individuals, housing and neighbourhoods. Areas were at times divided into sub-areas to assist with this process [24].

Thus, we weighted each case in turn by the following area characteristics:

1) Respondent's gender: male/female

2) Respondent's age group: 16-24/25-39/40-54/55-65/65+ years

3) Respondent's housing tenure: owned/social or private rented

4) Adult population size in study area: subareas within study areas

5) Adult population size in intervention area types (IATs): study areas within IATs

The frequencies of the two tenure types for households in each of the subareas were derived from the Glasgow City Council Tax Register. Populations of adults (16+ years old) in the study areas and intervention area types (further classified by gender and age group at subarea level) were estimated from NHS Community Health Index (CHI) records of GP registrations in the corresponding postcode units. Weights are the product of the five coefficients whose values correspond to the particular circumstances of each respondent.

In order that highly under-represented cases were not given excessive importance, weights were constrained to have a value of no more than five. Finally, all weights were multiplied by a constant so that the total number of weighted cases was equal to the actual number of interviews achieved.

### Data Analysis

Data analysis methods will be reported in the methods sections of future publications presenting the findings of specific analyses. Multi-level and individual level analysis will be employed as appropriate. Our primary interest is to examine changes in the circumstances, opinions and experiences of our respondent samples between survey waves. We compare change over time across the five IATs (rather than for individual study areas). However specific analyses may also focus on particular study areas or particular survey waves. For example, regression modelling is employed to explore potential causal relationships between intervention activity and health or social outcomes (i.e. to generate hypotheses for future analysis of change over time).

### Management of GoWell

The partners and sponsors in this collaboration maintain communication through quarterly Steering Group Meetings and informally through other contacts. The study staff have monthly team meetings and are sub-divided into groups that focus on specific components of the Programme. The principal investigators (currently AK, CT and LB; previously also MP and PH) share responsibility for overseeing the management and governance of the Programme. Fieldwork is conducted by GoWell researchers and contracted fieldworkers (via a commercial survey company).

### Ethics

GoWell's main programme of study (reported here) received ethical approval from NHS Scotland B MREC committee in 2005 (no. 05/MRE10/89). All participants gave written informed consent. In line with GoWell's ethics requirements and participant consents, sponsor organisations that are involved in intervention delivery (along with anyone else outside GoWell's research team) cannot access GoWell data except for publicly available anonymised findings. Data are recorded, transported and stored in accordance with data protection principals, ethical requirements and UK Medical Research Council guidance - for example, data that can identify individuals are physically locked away or electronically password protected, and are stored separately to research data with restricted access.

## Baseline results

### Response

For the baseline survey in 2006 contacts were attempted with 11,995 inhabited (as far as we could assess), residential addresses. A small number of households were removed from the sample for health and safety reasons (n = 25). Of the remaining 11,970 addresses, we achieved survey responses from 6,008. The response rate varied by area: the lowest area response rate was 39% and the highest was 65%. The overall baseline response rate was 50%.

The most common reason for non-response was inability to make contact with householders (38.5%), followed by refusal (10.5%). The response targets we set for fieldworkers were (approximately) achieved for LRAs, HIAs and PEs. The TRA target fell short due to non-responses and vacancies, so we used the opportunity to interview additional participants from the WSAs (see Table 3).

**Table 3:** GoWell baseline (2006) interview responses; summarised demographic and health characteristics of sample (unweighted).

	TRAs	LRAs	WSAs	HIAs	PEs	Total
**Response (n)**						
Target	1750	750	700	1400	1400	6000
Actual	1,435	726	1076	1371	1400	6008
**Gender (%)**						
Female	57.63	53.86	61.80	57.99	66.57	60.09
Male	42.37	46.14	38.20	42.01	33.43	39.91
**Age (%)**						
<25 years	11.57	8.95	5.86	3.36	9.00	7.76
25-39 years	42.37	35.95	20.82	15.32	24.64	27.43
40-54 years	23.41	25.21	28.44	22.03	29.57	25.65
55-64 years	7.04	10.74	13.48	18.16	16.57	13.40
65+ years	14.08	17.36	30.30	40.26	19.79	24.68
**Citizenship/ethnicity (%)**						
Scotland - white	53.12	62.82	97.16	96.70	96.18	82.16
Rest of UK - white	2.65	3.79	1.30	1.69	2.44	2.27
UK - black minority ethnic	4.50	4.21	1.30	0.88	0.22	2.03
Refugee	13.78	6.45	0.47	0.95	0.22	4.31
Asylum Seeker	24.69	17.95	0.19	0.29	0.43	8.05
**Household type (%)**						
Single parent family	20.22	14.98	9.95	6.37	15.39	13.46
Two parent family	24.23	21.50	17.18	10.55	19.76	18.47
Single adult	23.53	29.82	15.87	19.05	18.04	20.61
>1 adult	16.43	15.12	23.38	19.27	21.62	19.37
Pensioner	15.47	18.46	33.27	44.57	25.14	27.91
**Tenure (%)**						
Social rented	92.44	91.18	49.63	60.36	80.69	74.50
Private rented	2.47	0.96	2.70	2.55	1.36	2.09
Owner occupied	5.09	7.85	47.68	37.08	17.95	23.41
**Household employment status (%)**						
Employed	17.98	24.66	38.75	26.55	36.79	28.84
Not employed	65.16	55.23	26.86	28.01	37.79	42.24
Pensioner	15.47	18.46	33.27	44.57	25.14	27.91
**Current Smoker (%)**						
No	62.58	55.65	60.13	61.78	46.79	57.44
Yes	37.42	44.35	39.87	38.22	53.21	42.56
**GP visits in last 12 months (%)**						
None	25.57	28.55	20.00	17.88	16.21	21.00
1 or 2	26.62	30.34	36.84	27.45	42.29	32.74
3 or more	47.80	41.10	43.16	54.67	41.50	46.26
**Longstanding illness (%)**						
No	82.86	84.16	78.62	68.78	70.71	76.22
Yes	17.14	15.84	21.38	31.22	29.29	23.78
**Current general health: SF-12 (%)**						
Fair, Poor	20.48	25.48	21.47	28.08	26.00	24.28
Excellent, (very) good	79.51	74.52	78.53	71.70	74.00	75.67

### Sample characteristics

To provide an overview of our sample, Table 3 summarises a selection of unweighted baseline characteristics. Overall the respondents were predominantly female (60%); white Scottish (82%) and living in social rented accommodation (75%). Only a minority were in paid employment (29%). The sample contains a greater proportion of pensioners (>64 years old = 25%) compared to estimates for all Scotland (20%) and for all Glasgow (16%) [26]. The proportion of respondents who do not live with another adult (34%) is similar to the national estimate (33%) [27].

Twenty-four percent of GoWell participants reported their general health to be fair or poor (i.e. not 'good', as assessed by the SF-12): similar (one percentage point higher) to the national figure from the Scottish Health Survey (SHeS) 2003 [26]. GoWell respondents were less likely to report long standing illnesses compared to the corresponding SHeS figure (no long standing illness: GoWell = 76% (for men and for women); SHeS men = 60%; SHeS women = 58%) [28]. Smoking was more prevalent amongst GoWell respondents compared to Scotland as a whole (current smokers: GoWell men = 45%; GoWell women = 41%; SHeS men = 29%, SHeS women = 28%) [28]. Seventy-nine percent of GoWell respondents reported at least one GP consultation during the previous 12 months: similar to the national figure of 76% [29].

Baseline findings differ by intervention area type and are often patterned by the following groupings. Transformational Regeneration Areas and Local Regeneration Areas are similar types of neighbourhood (relatively poor post-war mass housing estates considered to need major investment). Wider Surrounding Areas and Housing Improvement Areas also have many similarities (they are more popular inner-city areas considered to need less investment). GoWell baseline data and routine data [14,30] for these intervention area types concur that TRA/LRA residents are more likely than WSA/HIA residents to be younger, belong to an ethnic minority group (particularly to asylum seeker and refugee communities), live in social rented homes, live alone or as single parents, and be unemployed. The latter area types include more older residents and owner occupiers. The Peripheral Estates, which have received substantial investment in the past, tend to occupy a middle ground between the two groupings described above: PEs share some characteristics of the TRAs/LRAs, some of the WSAs/HIAs, and some unique features. For example, Peripheral Estates tend to include a more even spread of age-groups, more people with long-standing illnesses and more smokers.

### Focus Groups

To date, focus group topics have included: community engagement, experiences of asylum seekers and refugees, the experience of housing improvement, transformational regeneration, being relocated in advance of demolition, and youth-related anti-social behaviour. Findings have been disseminated to stakeholder organisations as part of GoWell's formative evaluation.

## Discussion

This paper has provided an overview of the GoWell Programme and a more detailed description of the Community Health and Wellbeing Survey. GoWell is a mixed methods study that aims to improve our knowledge of the impacts of different approaches to regeneration on the health and wellbeing of residents in deprived urban areas. Compared to many housing improvement and regeneration studies it is large, long term and explores a range of different outcomes including health outcomes [5,31]. Its comparative design will help us better understand the impacts of different multi-faceted approaches to regeneration.

GoWell is intended to be 'theory generating' with regard to identifying mechanisms and pathways to health. GoWell's qualitative research components will help us understand what different stakeholders consider to be the most important aspects of regeneration and ways in which regeneration impacts upon their lives [32]. One early result of our work has been the development of a 'capitals framework' comprising six component capitals for understanding and investigating the impact of regeneration upon the physical, mental and social health of communities [4].

GoWell is of interest methodologically as a study that evaluates a social intervention affecting wider determinants of health, health inequalities and wellbeing. The regeneration of Glasgow would meet most definitions of a complex intervention [33,34]: it includes multiple, inter-related activities delivered by many different partners and is 'emergent' in that intervention plans change over time for a variety of reasons (and sometimes in response to findings from the GoWell study). GoWell recognises that our understanding of public health improvement can be enhanced by treating complex interventions such as regeneration as 'natural experiments'. The potential uses (and under-use) of this approach has been stated elsewhere [35].

### Challenges

One reason suggested for there being relatively few evaluations of the health effects of complex social interventions is that these evaluations are so difficult to conduct [34-37]. 'Difficult' in this context can mean resource intensive, requiring methodological compromises, multiple stakeholder engagement, and the challenge of studying a changing environment over which the researcher has limited control or no control.

For example, GoWell had to be designed and funded quickly - in the relatively short time span between researchers becoming aware of regeneration plans and the intervention being delivered. GoWell researchers have not been responsible for the planning or delivery of regeneration interventions and we could not assign (randomly or otherwise) individuals to different interventions. We could not identify appropriate 'no intervention' comparison areas because similarly deprived urban neighbourhoods within or outside Glasgow are unlikely to remain free of regeneration interventions during the ten year study period.

### Limitations

There are methodological limitations associated with some of these challenges. By comparing different neighbourhood populations, we risk the possibility that findings are confounded by contextual effects (e.g. neighbourhood specific characteristics that may affect outcomes). We will attempt to mitigate those risks by adjusting the data to take account of baseline population and health characteristics, and at times by comparing those intervention area types that had the most similar baseline characteristics (i.e. the LRAs and TRAs, and the WSAs and the HIAs). However, this will not 'solve' the problem of contextual effects, nor will it be possible to precisely measure the magnitude of that problem.

GoWell's community health and wellbeing component includes a repeat cross-sectional study. This type of design cannot measure change to individuals over time. It does measure change at an area level. It can tell us whether residents are more or less likely to highly rate their home, neighbourhood, community, health and wellbeing at different points in the regeneration process. Residential mobility may be an important driver for any changes we measure and so we will monitor this. GoWell also has a longitudinal component that will trace the health and wellbeing of individuals over time. This will focus particularly on the Transformational and Local Regeneration Areas and will be described in future publications.

GoWell focuses on neighbourhoods experiencing multiple interventions. The quantitative data we obtain may be better geared towards providing evidence of the overall impact of regeneration programmes rather than untangling the effects of individual interventions. To help gain insights into the impacts of specific interventions we will draw upon findings from GoWell's qualitative research and nested studies.

### Policy Relevance

Policy statements on regeneration often identify health improvement and reductions in health inequalities as aims and/or outcomes. The UK Department of Health has contributed to the funding of urban regeneration since the 1970s [4]. The report of the Scottish Government's Ministerial Task Force on Health Inequalities (*Equally Well*) recently highlighted the importance of broadly defined regeneration activities, and the role of GoWell in evaluating such activities, as a means of tackling social inequalities in health [3]. In short, governments assume that urban regeneration improves health. GoWell tests that assumption.

### Stakeholder Relevance

Residents, housing managers and other practitioners involved in delivering the interventions have made it clear that they value the regular feedback from GoWell to assess progress and inform future activities. Sometimes this involves producing GoWell reports for specific stakeholders. For example, residents of one area asked us to analyse data on respiratory health and other outcomes in their neighbourhood. The resulting report was then used to convince housing managers to change regeneration plans for that area so that health damaging dwellings could be demolished.

## Conclusion

We know little about the health effects of urban regeneration and housing improvement because many such interventions have been studied using weak study designs, or not studied at all, whilst robust studies have tended to focus on specific dimensions of housing improvement and health. This is a common problem for complex social interventions, even though such interventions may be costly and affect the health and wellbeing of large populations in unpredictable ways. We know least about the effects of those interventions that are most likely to influence the wider determinants of health--a problem described elsewhere as the "inverse evidence law" [38,39]. This 'evidence deficit' has been ascribed in part to the difficulty of conducting robust evaluations, particularly when the researchers have no direct input into intervention planning and delivery. The GoWell programme has been developed in such circumstances and aims to demonstrate that credible and useful evidence can be obtained from natural experiments to improve our understanding of how to maximise the benefits of urban regeneration.

## Competing interests

GoWell is a collaborative partnership between the Glasgow Centre for Population Health, the University of Glasgow, and the MRC/CSO Social and Public Health Sciences Unit. The programme's main sponsors are Glasgow Housing Association, the Scottish Government, NHS Health Scotland and NHS Greater Glasgow and Clyde. Some of the sponsors (e.g. Glasgow Housing Association and the Scottish Government) are also delivering interventions being evaluated by GoWell. Such sponsors are represented on GoWell's steering group and can suggest specific research topics and comment on non-academic dissemination of findings. GoWell's research team does not include anyone from the organisations delivering interventions being evaluated, nor has anyone from those organisations contributed to or commented upon this manuscript.

## Authors' contributions

All the authors have contributed to GoWell's study design and methods. ME led the writing of this manuscript. All the other authors have been involved in revising it critically for important intellectual content and have approved the final version.

## Pre-publication history

The pre-publication history for this paper can be accessed here:

http://www.biomedcentral.com/1471-2288/10/41/prepub

## Supplementary Material

Additional file 1 **GoWell's study components**. The various elements of GoWell (a mixed methods, multi-component study) are described. Click here for file

Additional file 2 **GoWell community health and wellbeing survey questions**. List of questions (and their sources) used in GoWell's 2006 baseline survey. Click here for file
