# Supplementary material for: Protocol for a mixed methods study investigating the impact of investment in housing, regeneration and neighbourhood renewal on the health and wellbeing of residents: the GoWell programme
Source: BMC Med Res Methodol. 2010 May 11;10:41. doi: 10.1186/1471-2288-10-41 (PMC2876178; doi:10.1186/1471-2288-10-41)
Supplement: Additional file 1 — GoWell's study components. The various elements of GoWell (a mixed methods, multi-component study) are described. [file 1471-2288-10-41-S1.DOC]

**GoWell’s study c**omponents.

| **Community health and wellbeing survey.**  The impacts of housing improvements and neighbourhood regeneration activities upon individuals, households and communities Investigated through a multi-site study involving four repeat resident surveys over the planned ten year period. The survey will be conducted in 14-15 communities across the city, distributed across five area intervention types: transformational regeneration areas; local regeneration areas; housing improvement areas; peripheral estates; and wider surrounding areas. The first survey conducted in 2006 involved 6,000 residents. Follow-up surveys will be repeated at approximately two-year intervals and involve a sample of around 5,000 households in each survey. Focus group research will also be carried out after each survey wave to provide more detailed qualitative evidence for various aspects of the survey and particular groups.  **GoWell longitudinal study.**  A longitudinal panel established to study the impacts of the disruption associated with regeneration upon residents, as well as to assess the extent to which people who ‘gain’ out of regeneration comprise those who no longer live in the regenerated neighbourhoods. The GoWell Longitudinal Study is focused on 6 regeneration areas and will comprise three different cohort groups: i) a ‘remainers’ cohort - i.e. those people who were interviewed in Wave 1 of the survey and are known to be still living in the same study area, ii) an ‘outmovers’ cohort - i.e. those people who move voluntarily or who are relocated out of regeneration areas, either permanently or temporarily, and iii) an ‘inmovers’ cohort of people who move into one of the regeneration areas. A longitudinal study of families will also be undertaken using qualitative research methods to gain a better understanding of the ‘lived realities’ of regeneration. This component focuses particularly on the Transformational and Local Regeneration Areas.  **Ecological study: monitoring change across Glasgow.**  This component involves monitoring changes relating to housing, health and other outcomes across Glasgow, so that the changes in the study areas can be looked at in the context of wider trends. It involves different elements which include: looking at the historical and policy background within which community regeneration is taking place; investigating the understandings and expectations of policy-makers and practitioners; secondary data analysis and data linkage for all neighbourhoods in the city; neighbourhood audits of the study areas; and the development of a housing taxonomy and health profiles in order to examine the links between housing types and health status across the city.  **Qualitative study of governance, empowerment and participation.**  A programme of focus groups and in-depth interviews with residents, policy-makers and practitioners will be undertaken in order to gain an understanding of how the governance of neighbourhood change is working out in practice. By working in several local areas and collecting different and sometimes competing perspectives on the effectiveness of partnership working, neighbourhood change and community engagement we will seek to identify those aspects of change most valued by residents and to suggest the most successful approaches to community engagement and empowerment.  **Evaluations of ‘wider action’ interventions and aspects of regeneration policy.**  This component allows for shorter-term ‘nested studies’ of specific initiatives aimed at improving particular aspects of communities or in-depth evaluations of certain policies or aspects of regeneration to be carried out. To date these ‘nested studies’ have included evaluations of youth projects, improvements to children’s playgrounds, and an environment and employability programme. |
| --- |
