# Supplementary material for: Protocol for a mixed methods study investigating the impact of investment in housing, regeneration and neighbourhood renewal on the health and wellbeing of residents: the GoWell programme
Source: BMC Med Res Methodol. 2010 May 11;10:41. doi: 10.1186/1471-2288-10-41 (PMC2876178; doi:10.1186/1471-2288-10-41)
Supplement: Additional file 2 — GoWell community health and wellbeing survey questions. List of questions (and their sources) used in GoWell's 2006 baseline survey. [file 1471-2288-10-41-S2.DOC]

**GoWell community health and wellbeing survey questions** (2006)

| **No.** | **QUESTION** | **SOURCE** |
| --- | --- | --- |
|  | **RESPONDENT’S NAME :**  **ADDRESS** : (Address Line 1)  (Address Line 2)  (Address Line 3)  (Postal Town)  **POSTCODE** : (ESSENTIAL)  **TELEPHONE**: (INCLUDING STD) (ESSENTIAL) |  |
| A | **SAMPLE AREA/SUB AREA** | GoWell* |
| B | **Which option best describes the home respondent lives in?** | GoWell* |
| C | **What floor does respondent live on?** | GoWell* |
| 1 | **Which of the following best describes your home?**  Rented from a private landlord  Rented from a family member, friend/acquaintance  Rented from Glasgow Housing Association or other housing association  Owned with a mortgage  Owned outright  Shared owner with Glasgow Housing Association or other housing association  Other, please specify | Adapted from Twenty-07* |
| 2 | **What is the name of your landlord?** | GoWell* |
| 3A | **I would now like you to think of everyone living in your household, including any children, How many people live here regularly as members of this household, please include yourself in this figure?** | GoWell* |
| 3A | **How many people live here including yourself?** | Adapted from SHARP* |
| 3B | **And is … male or female?** | Adapted from SHARP* |
| 3C | **Can you go through each household member and give their age last birthday – starting with yourself?** | Adapted from SHARP* |
| 3C | **Which of the following age bands apply?**  LESS THAN 16 YRS  16 – 17  18 – 24  25 – 39  40 – 54  55 – 64  65 PLUS  DON’T KNOW  REFUSED | GoWell* |
| 3D | **What is the relationship of each household member to you?** ONE CODE ONLY PER HH MEMBER  Spouse/partner/cohabite  Son/Daughter (including step/adopted)  Grandson/granddaughter (including step/adopted)  Parent/parent in-law  Other relative  Other non-relative | Adapted SHARP* |
| 3E | **Do any of the people you mentioned live away from the house most of the time?** | GoWell* |
| 3F | **Do you or does anyone in your household have any longstanding illness, disability or infirmity?** (longstanding means anything that has troubled you over a period of time or that is likely to affect you over a period of time) | Adapted from GHA Rehousing Survey 2005* |
| 3G | **Which of these best describes … current position?** ASK FOR ALL IN HH AGED 16+  ACCEPT ONLY ONE ANSWER PER HH MEMBER.  Full-time paid work (including self-employed)  Part-time paid work (includes self-employed)  Government or other training scheme  Unemployed  Retired  Temporary sick  Long term sick/disabled without a job  Looking after the home/family  Full time education  Other, specify | Adapted SHARP* |
| 3H | **And does anyone in your household do any …?** ASK FOR ALL IN HH AGED 16+ YRS   - **Voluntary work** - **OR Work as unpaid carer** | GoWell* |
| 4 | **How many rooms are there in your house not including bathrooms, kitchen, hall and toilets?** PROMPT IF NECESSARY. LIVING ROOMS THAT CONTAIN A SMALL KITCHEN AREA/KITCHENETTE ARE INCLUDED. DINING ROOMS AND STUDIES ARE INCLUDED. DINING KITCHENS ARE EXCLUDED. CELLARS AND LOFTS SHOULD BE EXCLUDED UNLESS THEY CONTAIN PUBLIC ROOMS OR BEDROOMS. WINDOWLESS ‘RECESS’ ROOMS ARE EXCLUDED. WRITE IN NUMBER | GoWell* |
| 5A | **How long in total have you lived at your home?** | Adapted from GHA Rehousing Survey 2005* |
| 5B | **How long in total have you lived in this area?** | Adapted from GHA Rehousing Survey 2005* |
| 6 | **Overall, how satisfied or dissatisfied are you with your home?** | Adapted from GHA Rehousing Survey 2005* |
| 7 | **Do you intend to move home in the next 12 months?** | Adapted from SHARP* |
| 8 | **Why do you intend to move house?**  DO NOT PROMPT. PROBE FULLY:  **For what other reasons do you intend to move?** CODE ALL THAT APPLY, WRITING IN ANY REASONS MENTIONED NOT INCLUDED IN THE LIST UNDER OTHER | Adapted from SHARP* |
| 9 | **Would you rate your current home in terms of the following?** |  |
| a | **Overall condition of home** | GoWell* |
| b | **Overall space** | Adapted GHA Social Survey 2005* |
| c | **Storage space** | GoWell* |
| d | **Quality or condition of bathroom/shower room** | Adapted GHA Social Survey 2005* |
| e | **Quality or condition of kitchen** | Adapted GHA Social Survey 2005* |
| f | **Heating system** | Adapted GHA Social Survey 2005* |
| g | **Insulation** | Adapted SHARP* |
| h | **State of repair inside the home** | GoWell* |
| i | **Internal decoration** | GoWell* |
| j | **State of repair outside the home for example roofs, gutters, walls** | GoWell/Adapted SHARP* |
| k | **External appearance** | Adapted GHA Social Survey 2005* |
| l | **Front door** | Adapted GHA Social Survey 2005* |
| m | **Security of the home** | Adapted GHA Social Survey 2005* |
| n | **Internal layout of home** | Adapted GHA Social Survey 2005* |
| o | **Windows** | Adapted GHA Social Survey 2005* |
| p | **Electrical wiring** | Adapted GHA Social Survey 2005* |
| q | **Access to a garden or somewhere to sit outside** | GoWell* |
| r | **Security of the common areas / close** | GoWell* |
| s | **Noise within the building** | Adapted GHA Social Survey 2005* |
| t | **Parking** | Adapted Scottish Household Condition Questionnaire 1996 [1] |
| 10 | **Which of these statements best sums up your views about your home?**  My home is fine as it is  My home needs some work to improve it  My home needs major work to improve it  My home needs to be demolished | GHA Social Survey 2005* |
| 11 | **In your opinion, what investment does your home need to improve it?**  CODE ALL THAT APPLY IN COLUMN Q11. WRITE IN ANY OTHER MENTIONS UNDER OTHER.  My home is fine as it is  My home needs some work to improve it  My home needs major work to improve it  My home needs to be demolished | GoWell, adapted from GHA Social Survey 2005 and SHARP* |
| 12 | **Have any improvements been carried out to your home in the last 12 months?**  Extension  Conversion  Complete refurbishment  Adaptations for people with disability  Roof, chimneys and gutters  Outside walls, damp proof course  External doors, windows  Floors, stairs  Ceilings, inside walls, doors  Gas, water, electricity  Foundations, drains  Bathroom units  Kitchen units  Heating  Insulation  Home safety/security measures  Other, write in  No opinion/don’t know | GoWell * |
| 13 | **Which of these improvements has your home had completed in the last 12 months?** | GoWell, adapted from GHA Social Survey 2005 and SHARP* |
| 14 | **Generally how satisfied or dissatisfied are you with the improvement work that has been carried out on your home?** | GoWell * |
| 15 | **Thinking about your landlord/factor (as applicable), how satisfied or dissatisfied are you with each of the following…?**  ASK Q15 AMONGST THOSE RESPONDENTS WHO HAVE A LANDLORD/FACTOR (Q1 CODE 3 OR 6), OTHERWISE GO TO Q16.  *SHOWCARD 5* –  READ OUT. SINGLE CODE ONLY | Adapted GHA Social Survey 2005* |
| a | **The way you are kept informed about things that might affect you** | Adapted GHA Social Survey 2005* |
| b | **Their willingness to take account of residents’ views when making decisions** | GoWell* |
| 16 | **How much do you agree or disagree with the following statements?** | SHARP* |
| a | **I feel I have privacy in my home** | SHARP* |
| b | **I feel in control of my home** | SHARP* |
| c | **My home makes me feel that I’m doing well in my life** | SHARP* |
| d | **I feel safe in my home** | Adapted SHARP * |
| e | **I can get away from it all in my home** | SHARP* |
| 17 | READ OUT:  **Now I am going to ask you about your local neighbourhood. By neighbourhood, I mean the local area within a 5 to 10 minutes walk from your home.**  *SHOWCARD 5* –  **How satisfied or dissatisfied are you with this neighbourhood as a place to live?**  SINGLE CODE ONLY. | Adapted from Survey Of English Housing 2000-1[2] |
| 18 | **How much do you agree or disagree with the following statements?** | GoWell * |
| a | **Living in this neighbourhood helps make me feel that I’m doing well in my life** | GoWell * |
| b | **Many people in Glasgow think this neighbourhood has a bad reputation** | GoWell * |
| c | **People who live in this neighbourhood think highly of it** | GoWell * |
| d | **On your own, or with others, you can influence decisions affecting your local area** | Home Office Citizenship Survey 2001[3] |
| 19 | **For each of the following statements, could you tell me whether you think that each of these is a serious problem** | Adapted GHA Social Survey 2005 and SHARP* and Scottish Household Condition Questionnaire 1996[1] |
| a | **Vandalism, graffiti and other deliberate damage to property or vehicles** | Adapted GHA Social Survey 2005 and SHARP* and Scottish Household Condition Questionnaire 1996[1] |
| b | **Violence including assaults and muggings** | Adapted from SHARP* |
| c | **People being insulted, pestered or intimidated in the street** | Adapted from SHARP* |
| d | **Noisy neighbours or loud parties** | GHA Social Survey 2005* |
| e | **Abandoned or burnt out cars** | Adapted GHA Social Survey 2005* |
| f | **People being attacked or harassed because of their skin colour, ethnic origin** | Adapted GHA Social Survey 2005* |
| g | **People using or dealing drugs** | Adapted GHA Social Survey 2005 and SHARP* |
| h | **People being drunk or rowdy in public places** | Adapted GHA Social Survey 2005 and SHARP* |
| i | **Gang activity** | GoWell* |
| j | **Teenagers hanging around on the street** | Adapted GHA Social Survey 2005 and SHARP* |
| k | **Nuisance neighbours or problem families** | Adapted GHA Social Survey 2005 * and Scottish Household Condition Questionnaire 1996[1] |
| l | **Dogs roaming about / dog fouling / barking** | Adapted from SHARP* |
| m | **Rubbish or litter lying around** | Adapted GHA Social Survey 2005 and SHARP* |
| n | **Vacant or derelict buildings and sites** | Adapted from Scottish Social Attitudes Survey 2004[4] |
| o | **Tensions between Protestants and Catholics** | GoWell* |
| p | **House break-ins / burglary** | Adapted GHA Social Survey 2005 and SHARP* |
| q | **Untidy gardens** | Adapted GHA Social Survey 2005* |
| 20 | **How would you rate the quality of your neighbourhood in terms of the following things…?** |  |
| a | **Attractive buildings** | Adapted from Scottish Household Condition Questionnaire 1996[1] |
| b | **Attractive environment** | Adapted from Scottish Household Condition Questionnaire 1996[1] |
| c | **Quiet and peaceful environment** | Adapted from Scottish Household Condition Questionnaire 1996[1] |
| d | **Park / open spaces** | Adapted from Scottish Household Condition Questionnaire 1996[1] |
| e | **Children’s play area** | Adapted from Scottish Household Condition Questionnaire 1996[1] |
| 21 | **How safe would you feel walking alone in this neighbourhood after dark?**  SINGLE CODE ONLY. IF RESPONDENT STATES THAT NEVER WALKS ALONE AFTER DARK THEN PROMPT:  **Is that because you don’t feel safe?**  IF YES PROBE:  **Would you say you feel a bit unsafe or very unsafe?** CODE ACCORDINGLY  Very safe  Fairly safe  Neither safe nor unsafe  A bit unsafe  Very unsafe  DO NOT PROMPT: Never walk alone after dark | Adapted Home Office Citizenship Survey 2001[3] |
| 22 | **To what extent do you feel that you belong to this neighbourhood?** | Adapted from British Home Office Citizenship Survey[3] |
| 23 | **To what extent do you agree that this neighbourhood is a place where people from different backgrounds get on well together?** | Adapted from Home Office Citizenship Survey[3] |
| 24 | **How would you rate the quality of the following services in and around your local area?** |  |
| a | **Schools** | Adapted from NDC* , Survey Of English Housing [2], General Household Survey 2001[5] |
| b | **Public Transport** | Adapted from Scottish Social Attitudes Survey 2004[4], Survey Of English Housing[2], General Household Survey 2001[5] |
| c | **Rubbish Collection** | Adapted from General Household Survey 2001 [5] |
| d | **Youth and leisure services** | Adapted from Survey Of English Housing [2], General Household Survey 2001 [5] |
| e | **Policing** | Adapted from General Household Survey 2001 [5] |
| f | **Shops** | Adapted from Scottish Household Condition Questionnaire 1996[1], Survey Of English Housing[2] |
| g | **Banking or financial services** | Adapted from Survey Of English Housing[2] |
| h | **Childcare or nurseries** | Adapted from NDC* |
| i | **Health centre / GP** | Adapted from Survey Of English Housing[2] , General Household Survey 2001 [5] |
| 25 | **For each of the following types of places or amenities that people go to, can you tell me whether you mostly use these amenities within or outside your local area?** |  |
| a | **Sport facilities, swimming pool or gym** | Adapted from SHARP* |
| b | **Other social venues (e.g. bingo, pub, bowling, dancing, social club)** | Adapted from SHARP* |
| c | **Park or play area** | Adapted from SHARP* |
| d | **Post office** | Adapted from SHARP* |
| e | **Small/local grocers** | Adapted from SHARP* |
| f | **Supermarket** | Adapted from SHARP* |
| g | **General shopping (not food)** | GoWell* |
| h | **Library** | Adapted from SHARP* |
| i | **Community Centre** | GoWell* |
| j | **Job Centre** | GoWell* |
| 26 | **On the whole, do you think that over the past two years, this area has got better or worse to live in or has it stayed the same** | Adapted from NDC and ONS Social Capital Module [6]* |
| 27 | **For what reasons do you think the area has got better?**  ASK Q27 AMONGST THOSE RESPONDENTS WHO FELT THE AREA HAS GOT BETTER (Q26 CODE 1). PROBE FULLY:  **What else?**  WRITE IN RESPONSE VERBATIM | Adapted from NDC and ONS Social Capital Module[6] * |
| 28 | **For what reasons do you think the area has got worse?**  ASK Q28 AMONGST THOSE RESPONDENTS WHO FELT THE AREA HAS GOT WORSE (Q26 CODE 3). PROBE FULLY.  **What else?**  WRITE IN RESPONSE VERBATIM. | Adapted from NDC* |
| 29 | **Thinking about how often you personally contact your relatives, friends and neighbours but not counting the people you live with – how often do you do any of the following?** |  |
| a | **Meet up with relatives** | Adapted from ONS Social Capital Module[6], SHARP * |
| b | **Speak to relatives on the phone** | Adapted from ONS Social Capital Module[6], SHARP * |
| c | **Write to relatives (including letters, texting, email and internet)** | Adapted from ONS Social Capital Module[6], SHARP * |
| d | **Meet up with friends** | Adapted from ONS Social Capital Module[6], SHARP * |
| e | **Speak to friends on the phone** | Adapted from ONS Social Capital Module[6], SHARP * |
| f | **Write to friends (including letters, texting, email and internet)** | Adapted from ONS Social Capital Module[6], SHARP * |
| g | **Speak to neighbours** | Adapted from ONS Social Capital Module[6], SHARP * |
| 30 | **Thinking now about your relatives, friends and neighbours outside your home, can you tell me around how many people could you ask for the following kinds of help?** |  |
| a | **To go to the shop for messages if you are unwell** | Adapted from ONS Social Capital Module[6], SHARP, NDC* |
| b | **To lend you money to see you through the next few days** | Adapted from ONS Social Capital Module[6], SHARP, NDC* |
| c | **To give you advice and support in a crisis** | Adapted from ONS Social Capital Module[6], SHARP, NDC* |
| 31 | **Over the past 12 months, have you taken part in, supported or helped any groups, clubs or organisations?** | Adapted from ONS Social Capital Module[6], SHARP, NDC* |
| 32 | **To what extent do you agree or disagree with the following statements…** |  |
| a | **It is likely that someone would intervene if a group of youths were harassing someone in the local area** | Adapted from Twenty-07* |
| b | **People out of work should do more to find employment** | GoWell* |
| c | **Parents should take more responsibility for the behaviour of teenage children** | GoWell* |
| d | **People should take more responsibility for their own health** | GoWell* |
| e | **Immigrants improve Scottish society by bringing in new ideas and culture** | GoWell* |
| f | **People round here should do more to help the police** | GoWell* |
| g | **Someone who lost a purse or wallet around here would be likely to have it returned without anything missing** | Adapted from Home Office Citizenship Survey[3] |
| 33 | **I am now going to ask you some questions about your health and your general well-being. I would just like to remind you that all the information you give in this and the other sections of this questionnaire will be treated completely confidentially.**  **I’ll begin by asking how your health is now. Please try to answer the following questions as accurately as you can.**  **In general, would you say your health is…?**  Excellent  Very good  Good  Fair  or Poor | SF-12 V.2* |
| 34 | **Now I’m going to read out a couple of activities that you might do during a typical day. As I read each item, please tell me if your health limits you a lot, limits you a little, or does not limit you at all in these activities.**  READ ACTIVITY AND SAY:  **Does your health now limit you a lot, a little or not at all?**  SINGLE CODE ONLY FOR EACH. INTERVIEWER NOTE: IF CLAIM NOT TO DO ACTIVITY THEN ASK: **Is this because of your health?**  IF YES, PROBE FOR WHETHER HEALTH LIMITS A LOT OR A LITTLE AND CODE ACCORDINGLY. | SF-12 V.2* |
| a) | moderate activities such as moving a table, pushing a vacuum cleaner, bowling or playing golf | SF-12 V.2* |
| b) | climbing several flights of stairs | SF-12 V.2* |
| 35 | READ OUT: I’m now going to ask you about your physical and emotional health in the past four weeks and the effect of this on your daily activities.  *SHOWCARD 18* –During the past four weeks how much of the time have you …. ASK a. to d., DO NOT ROTATE ORDER OF ASKING. SINGLE CODE ONLY FOR EACH | SF-12 V.2* |
| a | **Accomplished less than you would like as a result of your physical health** | SF-12 V.2* |
| b | **Been limited in the kind of work or other regular daily activities you do as a result of your physical health** | SF-12 V.2* |
| c | **Accomplished less than you would like as a result of any emotional problems, such as feeling depressed or anxious** | SF-12 V.2* |
| d | **Done work or other regular daily activities less carefully than usual as a result of any emotional problems, such as feeling depressed or anxious** | SF-12 V.2* |
| 36 | **Still thinking about the past four weeks, how much did pain interfere with your normal work, including both work outside the home and housework?** | SF-12 V.2* |
| 37 | READ OUT:  **The next questions are about how you feel and how things have been with you during the past four weeks. As I read each statement, please give me the one answer that comes closest to the way you have been feeling; is it all of the time, most of the time, some of the time, a little of the time or none of the time**?  *SHOWCARD 19*.  #  **How much of the time during the past four weeks (have you) …?**  READ STATEMENTS a. to d.. DO NOT ROTATE ORDER OF ASKING. SINGLE CODE ONLY FOR EACH. | SF-12 V.2* |
| a | **Felt calm and peaceful** | SF-12 V.2* |
| b | **Had a lot of energy** | SF-12 V.2* |
| c | **Felt downhearted and depressed** | SF-12 V.2* |
| d | **Has your physical health or emotional problems interfered with your social activities like visiting friends, relatives** | SF-12 V.2* |
| 38 | **Within the last four weeks, have you suffered from any of the problems listed on this card?** | [Health and Lifestyle Survey 1991 - Main Questionnaire (Part 2)](http://qb.soc.surrey.ac.uk/surveys/hals/hals91que2.pdf#xml=http://qb.soc.surrey.ac.uk/cgi-bin/semaphore/pdf_hl?STEMMER=en&RGB=ff00ff&WORDS=palpitations%20sinus%20breathless%20troubl&ALL=&ANY=Sleeplessness%20Palpitations%20or%20breathlessness%20Sinus%20trouble%20&EXACTB=0&PHRASE=&EXACTP=0&DB=questionbank&URL=/surveys/hals/hals91que2.pdf) [7] |
|  | **Sleeplessness** | Adapted Health and Lifestyle Survey 1991 - Main Questionnaire (Part 2) [7] |
|  | **Palpitations or breathlessness** | Health and Lifestyle Survey 1991 - Main Questionnaire (Part 2)[7] |
|  | **Sinus trouble or catarrh** | Health and Lifestyle Survey 1991 - Main Questionnaire (Part 2)[7] |
|  | **Persistent cough** | Health and Lifestyle Survey 1991 - Main Questionnaire (Part 2)[7] |
|  | **Faints/dizziness** | Health and Lifestyle Survey 1991 - Main Questionnaire (Part 2)[7] |
|  | **Pain in chest** | Adapted Health and Lifestyle Survey 1991 - Main Questionnaire (Part 2) [7] |
|  | **Migraines or frequent headaches** | Adapted Health and Lifestyle Survey 1991 - Main Questionnaire (Part 2)[7] |
|  | **Difficulty walking** | Adapted Health and Lifestyle Survey 1991 - Main Questionnaire (Part 2)[7] |
|  | **Any other pain (PLEASE SPECIFY WHERE)** | Adapted Health and Lifestyle Survey 1991 - Main Questionnaire (Part 2)[7] |
|  | **None of these** | Health and Lifestyle Survey 1991 - Main Questionnaire (Part 2)[7] |
| 39 | READ OUT:  **I’d now like you to think about your health over the past year.**  **Over the last 12 months, would you say your health has on the whole been…?**  READ RESPONSE LIST. SINGLE CODE ONLY | Census for Scotland[8] |
| 40 | **Do you have any of the following health problems or disabilities listed on this card?** |  |
|  | **Deafness /severe hearing impairment /difficulty in hearing** | Adapted British Household Panel Survey 2004[9] |
|  | **Blindness /severe vision impairment/difficulty in seeing (other than needing glasses to read normal size print)** | Adapted British Household Panel Survey 2004[9] |
|  | **Problems or disability connected with: arms, legs, hands, feet, back or neck (including arthritis and rheumatism)** | British Household Panel Survey 2004[9] |
|  | **A condition that substantially limits one or more basic physical activities such as walking, climbing stairs, lifting or carrying** | Test Census[8] |
|  | **Skin conditions/allergies** | British Household Panel Survey 2004[9] |
|  | **Breathing problems/asthma/bronchitis** | Adapted British Household Panel Survey 2004[9] |
|  | **Heart/high blood pressure/blood circulation problems** | British Household Panel Survey 2004[9] |
|  | **Stomach/liver/kidney/digestive problems** | British Household Panel Survey 2004[9] |
|  | **Diabetes** | British Household Panel Survey 2004[9] |
|  | **Epilepsy** | British Household Panel Survey 2004[9] |
|  | **Migraine or frequent headaches** | British Household Panel Survey 2004[9] |
|  | **Cancer** | British Household Panel Survey 2004[9] |
|  | **Stroke** | British Household Panel Survey 2004[9] |
|  | **Infections** | Test Census[8] |
|  | **A learning difficulty** | Test Census[8] |
|  | **A psychological or emotional condition** | Adapted British Household Panel Survey 2004[9] |
|  | **Other health problems (PLEASE SPECIFY WHICH)** | British Household Panel Survey 2004[9] |
|  | **None of these** | GoWell* |
| 41 | **In the past 12 months, how many times have you seen or spoken to a doctor from your practice regarding your own health or wellbeing?** | Adapted from SHARP* |
| 42 | **And in the past 12 months, have you spoken to a GP or family doctor on your own behalf, either in person or by telephone about being anxious or depressed or about a mental, nervous or emotional problem (including stress)?** | Adapted from Health Survey for England 2001[10] |
| 43 | **I would now like to ask you some questions about things that may affect your health, like smoking, drinking and life-style. Firstly, smoking:**  **Do you, or have you ever, smoked?**  SINGLE CODE ONLY.  INTERVIEWER NOTE: IF ASKED THIS REFERS TO ANY KIND OF TOBACCO SMOKING INCLUDING CIGARETTES, ROLL UPS, PIPE TOBACCO OR CIGARS  I smoke daily  I smoke occasionally now but not every day  I’ve smoked in the past but not now  I’ve never smoked | [Adapted from Scottish Health Survey 2003](http://qb.soc.surrey.ac.uk/surveys/ghs/03individual.pdf#xml=http://qb.soc.surrey.ac.uk/cgi-bin/semaphore/pdf_hl?STEMMER=en&RGB=ff00ff&WORDS=i%20intend%20to%20give%20up%20smoke%20within%20the%20next%20month&ALL=&ANY=&EXACTB=0&PHRASE=I%20intend%20to%20give%20up%20smoking%20within%20the%20next%20month&EXACTP=0&DB) [11] |
| 44 | IF Q43 CODE 1 ASK:  **How many cigarettes do you smoke per day?**  IF NECESSARY PROMPT:  **A roll up is to be considered as a cigarette.**  IF Q43 CODE 2 ASK:  **How many cigarettes do you smoke per week?**  IF NECESSARY PROMPT:  **A roll up is to be considered as a cigarette.** | [Adapted from Scottish Health Survey 2003](http://qb.soc.surrey.ac.uk/surveys/ghs/03individual.pdf#xml=http://qb.soc.surrey.ac.uk/cgi-bin/semaphore/pdf_hl?STEMMER=en&RGB=ff00ff&WORDS=i%20intend%20to%20give%20up%20smoke%20within%20the%20next%20month&ALL=&ANY=&EXACTB=0&PHRASE=I%20intend%20to%20give%20up%20smoking%20within%20the%20next%20month&EXACTP=0&DB) [11] |
| 45 | **Which of the following phrases on this card best describes your future smoking habits?**  I intend to give up smoking within the next month  I intend to give up smoking within the next 6 months  I intend to give up smoking within the next year  I intend to give up smoking but not in the next year  I intend to give up smoking but I’m not sure when  I don’t intend to give up smoking | [Adapted General Household Survey 2003 - Individual Questionnaire](http://qb.soc.surrey.ac.uk/surveys/ghs/03individual.pdf#xml=http://qb.soc.surrey.ac.uk/cgi-bin/semaphore/pdf_hl?STEMMER=en&RGB=ff00ff&WORDS=i%20intend%20to%20give%20up%20smoke%20within%20the%20next%20month&ALL=&ANY=&EXACTB=0&PHRASE=I%20intend%20to%20give%20up%20smoking%20within%20the%20next%20month&EXACTP=0&DB) [12] |
| 46 | **Do you, or have you ever, drunk alcoholic drinks?** | Adapted from SHARP* and Scottish Health Survey 2003[11] |
| 47 | **Thinking about an average week, approximately how much would you drink of each of the following … ?**  Number of pints normal strength lager/beer/cider  Number of bottles of normal strength lager/beer/cider  Number of cans of normal strength lager/beer/cider  Number of pints of strong lager/beer/cider  Number of bottles of strong lager/beer/cider  Number of cans of strong lager/beer/cider  Number of glasses of wine (1 bottle = 6 glasses)  Number of bottles of alcopops  Number of spirit measures | Provided By BMG* |
| 48 | **Have you used any recreational drugs in the last four weeks? By this we mean drugs such as cannabis, cocaine, ecstasy, heroin, magic mushrooms, or tranquillizers if they are not being taken on a doctor’s prescription.** | Adapted from SHARP*. |
| 49 | **I would now like to ask you some questions about the food you eat.**  **On average, how many portions of the following do you eat each day?** | Adapted from SHARP* |
| a | **Fruit whereby one portion is one apple, one tomato, 2 tablespoons of canned fruit, one small glass of fruit juice** | Adapted from SHARP*. |
| b | **Vegetables or salad (not potatoes) whereby one portion is two tablespoons** | Adapted from SHARP*. |
| 50 | **In the last 7 days, how many times did your main meal of the day come from a takeaway or fast-food seller of some kind?** | GoWell* |
| 51 | **In a typical week, on how many days do you do each of the following activities?** |  |
| a | **30 minutes of moderate physical exercise such as brisk walking, cleaning the house – it doesn’t have to be 30 minutes all at once** | Adapted Scottish Health Survey 2003[11] |
| b | **20 continuous minutes doing vigorous exercise, enough to make you sweaty and out of breath such as fitness workout or some kind of physical work** | Adapted Scottish Health Survey 2003[11] |
| c | **Go for a walk around the neighbourhood** | GoWell* |
| 52 | **What is your weight?** | GoWell* |
| 53 | **What is your height?** | GoWell* |
| 54 | **We would now like you to think about your children who are less than 17 years old.**  *SHOWCARD 24* –  **In the past 12 months, how many of the children in your household have been seen by a doctor or treated for each of the following conditions?**  WRITE IN NUMBER OF CHILDREN THAT HAVE BEEN TREATED FOR EACH CONDITION | Adapted from SHARP* |
|  | **1. Asthma** | Adapted from SHARP* |
|  | **2. Eczema** | Adapted from SHARP* |
|  | **3. Bronchitis** | Adapted from SHARP* |
|  | **4. Headaches** | Adapted from SHARP* |
|  | **5. Allergies** | Adapted from SHARP* |
|  | **6. Sleeping problems** | Adapted from SHARP* |
|  | **7. Sinus/Catarrh** | Adapted from SHARP* |
|  | **8. Digestive problems** | Adapted from SHARP* |
|  | **9. Bed wetting** | GoWell* |
|  | **10. Persistent cough** | Adapted from SHARP* |
|  | **95. Other long term disability or illness, write in** | Adapted from SHARP* |
|  | **97. Don’t know** | Adapted from SHARP* |
|  | **98. Refused** | Adapted from SHARP* |
| 55 | **Can I just confirm how many children you have aged under 17 years that are in full time education?** | GoWell* |
| 56 | **Approximately how many days off school have these children had in total in the last month (excluding weekends and holidays)? Write in one single figure totalling all the days that all children had off school** | Adapted from SHARP* |
|  | REPEAT OF QUESTION Q3g | Standard Occupational Classification [13] |
| 57a | **What is your job title e.g. assistant chef?** | Standard Occupational Classification [13] |
| 57b | **What do you mainly do in your job?** PROBE FULLY. (MAIN JOB IF MORE THAN ONE) | Standard Occupational Classification [13] |
| 58 | **How long is it since you last had a paid job of at least 16 hours a week (excluding holiday jobs)?** | Standard Occupational Classification [13] |
| 59a | **What is the job title for the main job that you did e.g. assistant chef?** | Standard Occupational Classification [13] |
| 59b | **What did you mainly do in your job?**  PROBE FULLY. (MAIN JOB IF MORE THAN ONE) | Standard Occupational Classification [13] |
| 60 | **Which of the following best describes your current or last job?**  Self employed with paid employees  Self employed with NO paid employees  Manager  Supervisor  Other employee | Standard Occupational Classification [13] |
| 61 | **How many people are employed where you work or last worked, under 25 staff or over 25?** | Standard Occupational Classification [13] |
| 62 | **How satisfied or dissatisfied are you with your employment situation at the moment?** | GoWell * |
| 63 | **Does your household currently receive housing benefits to pay for the rent/mortgage?** | Adapted from GHA Social Survey *2005 |
| 64 | **Which of the letters on this card represents the total income of your *HOUSEHOLD* from all sources before tax – including benefits, savings and so on? Please just tell me the letter.**  SINGLE CODE ONLY.  *(LETTERS RELATE TO LIST OF INCOME BANDS ON SHOWCARD)* | Adapted from GHA Social Survey 2005* |
| 65A | **Would you be able to tell me whether the total annual income of your household from all sources before tax – including benefits, savings and so on is more or less than £20,300 (or £391 weekly)?** | GoWell* |
| 65B | PROBE:  **And would you mind telling me if your total annual income is less than £10,150 (or £196 weekly)?** | GoWell* |
| 66 | **Which of these statements best describes you (and your partner’s) income excluding housing benefit or child benefit?**  Wholly from state benefits or pensions  Partly from state benefits or pensions  Earning or private income  Don’t know  Rather not say | Adapted from GHA Social Survey 2005* |
| 67 | **Looking at the card, which option best describes how often you find it difficult to meet the cost of the following** | Adapted from GHA Social Survey 2005* |
| a | **Rent or mortgage** | Adapted from SHARP* |
| b | **Repairs, maintenance or factor charges for your home** | Adapted from SHARP* |
| c | **Gas, electricity and other fuel bills** | Adapted from SHARP* |
| d | **Food** | Adapted from SHARP* |
| e | **Council tax** | Adapted from SHARP* |
| 68 | **Over the past 12 months have you or your partner (if applicable) used any these ways to borrow money?** |  |
|  | **Bank overdraft** | Adapted from NDC* |
|  | **Fixed term loan from Bank or Building society (not a mortgage)** | Adapted from NDC* |
|  | **Loan from a credit union** | Adapted from NDC* |
|  | **Loan from an insurance/finance company** | Adapted from NDC* |
|  | **Loan from a home credit provider, like Provident** | Adapted from NDC* |
|  | **Loan from a money lender or ‘tally’ man** | Adapted from NDC* |
|  | **Loan from a friend or relative** | Adapted from NDC* |
|  | **Loan or advance on wages from employer** | Adapted from NDC* |
|  | **DSS or Social Fund loan** | Adapted from NDC* |
|  | **Cheque cashing service where money is not taken from your account for a week or two** | Adapted from NDC* |
|  | **Loan from a community bank** | Adapted from NDC* |
|  | **Pawn broker/Cash converter/sale of personal goods** | Adapted from NDC* |
|  | **Used savings** | Adapted from NDC* |
|  | **Other (please specify)** | Adapted from NDC* |
|  | **None of these** | Adapted from NDC* |
|  | **Don’t know/can’t remember** | Adapted from NDC* |
|  | **Refused** | Adapted from NDC* |
| 69 | **Can you tell me what is the highest level of educational qualifications you’ve obtained?** |  |
|  | **School leaving certificate** | Adapted from various – Eg, SHARP, NDC, GHA Etc* |
|  | **O Grade, Standard Grade, GCSE, CSE, or equivalent – grades D to F** | Adapted from various – Eg, SHARP, NDC, GHA Etc* |
|  | **O Grade, Standard Grade, GCSE, CSE, or equivalent – grades A to C** | Adapted from various – Eg, SHARP, NDC, GHA Etc* |
|  | **Higher Grade/A Level, AS Level, Advanced Senior cert, CSYS or equivalent** | Adapted from various – Eg, SHARP, NDC, GHA Etc* |
|  | **GSVQ or SVQ Level 1 or 2, BTEC First Diploma, City and Guilds Craft or equivalent** | Adapted from various – Eg, SHARP, NDC, GHA Etc* |
|  | **GSVQ or SVQ Level 3, ONC, OND or SCOTVEC National Diploma, City and Guilds Advanced Craft, RSA Advanced Diploma or equivalent** | Adapted from various – Eg, SHARP, NDC, GHA Etc* |
|  | **Apprenticeships or trade qualification** | Adapted from various – Eg, SHARP, NDC, GHA Etc* |
|  | **HNC, HND, SVQ levels 4 or 5, RSA Higher Diploma or equivalent** | Adapted from various – Eg, SHARP, NDC, GHA Etc * |
|  | **First degree, Higher Degree** | Adapted from various – Eg, SHARP, NDC, GHA Etc* |
|  | **Other technical or business qualification / certificate** | Adapted from various – Eg, SHARP, NDC, GHA Etc * |
|  | **OTHER** | Adapted from various – Eg, SHARP, NDC, GHA Etc * |
|  | **None of these** | Adapted from various – Eg, SHARP, NDC, GHA Etc * |
| 70 | **What is your current religion, denomination, body or faith?** | BMG adapted from various* |
|  | **No religion** | BMG adapted from various* |
|  | **Christian – no denomination** | BMG adapted from various* |
|  | **Roman Catholic** | BMG adapted from various* |
|  | **Church of England/Anglican/Episcopal** | BMG adapted from various* |
|  | **Presbyterian – Church of Scotland** | BMG adapted from various* |
|  | **Free Presbyterian** | BMG adapted from various* |
|  | **Methodist – including Wesleyan** | BMG adapted from various* |
|  | **Baptist** | BMG adapted from various* |
|  | **United Reformed Church/Congregational** | BMG adapted from various* |
|  | **Brethren** | BMG adapted from various* |
|  | **Other Protestant** | BMG adapted from various* |
|  | **Other Christian** | BMG adapted from various* |
|  | **Jewish** | BMG adapted from various* |
|  | **Hindu** | BMG adapted from various* |
|  | **Islam/Muslim** | BMG adapted from various* |
|  | **Sikh** | BMG adapted from various* |
|  | **Buddhist** | BMG adapted from various* |
|  | **Other non-Christian** | BMG adapted from various* |
|  | **Refused** | BMG adapted from various* |
| 71 | **To which of these groups do you consider you belong?**  **(LIST OF ETHNIC GROUPS)** | BMG adapted from various* |
| 72 | **Which of the categories on this list best describes your current situation?** | BMG adapted from various* |
|  | **British Citizen born in the UK** | BMG adapted from various* |
|  | **British Citizen born outside the UK** | BMG adapted from various* |
|  | **Or do any of the following describe your status in the UK** | BMG adapted from various* |
|  | **Indefinite leave to remain** | BMG adapted from various* |
|  | **Exceptional leave to remain** | BMG adapted from various* |
|  | **Or do any of the following describe your status in the UK** | BMG adapted from various* |
|  | **Appealing a refused asylum application/Judicial review pending** | BMG adapted from various* |
|  | **Received final refusal** | BMG adapted from various* |
|  | **OTHER** | BMG adapted from various* |
|  | **NO ANSWER** | BMG adapted from various* |
| 73 | **Can you tell me what your marital or civil partnership status is at present?** | BMG adapted from various* |
| 74 | **Do you own or have regular access to a car or van?** | Adapted from SHARP* and various Eg British Household Panel Survey 2000 |

# *Note: SHARP = Scotland's Housing And Regeneration Project (2002-2008)[14]; NDC = National Evaluation Of New Deal For Communities Household Survey Questionnaire Final [15]; Twenty-07 = The West of Scotland Twenty-07 Study[16-17]; GHA = Glasgow Housing Association; SF-12 v.2 = Short Form 12 Version 2[18]; GoWell = questions created by the GoWell team; BMG = questions supplied by BMG research, the market research company contracted to conduct the survey fieldwork. Also note that we have identified the sources of questions we used to populate the GoWell Questionnaire but it is possible that some questions may have appeared or originated in other surveys besides those listed in the above table. Survey questionnaires were located online (e.g. using Survey Question Bank: <http://surveynet.ac.uk/sqb/introduction.asp>), or through direct contact with researchers.

References

1. **Scottish Household Condition Survey Questionnaire** [http://surveynet.ac.uk/sqb/qb/surveys/shcs/96mainques.pdf]

2. National Centre for Social Research: **Survey of English Housing, 2000-2001**. London; 2004.

3. Attwood C, Singh G, Prime D, Creasey R: **2001 Home Office Citizenship Survey: people, families and communities**. In *Home Office Research Study. Volume 270*. London: Home Office; 2003.

4. Anderson S, Bromley C, Given L, Scottish Centre for Social Research: **Public attitudes towards young people and youth crime in Scotland: findings from the 2004 Scottish Social Attitudes Survey.** . Edinburgh: Nuffield Foundation, Scottish Government; 2006.

5. Office for National Statistics SSD: **Living in Britain: results from the 2001 General Household Survey**. London; 2002.

6. Babb P: **Measurement of social capital in the UK**. In *Social and Welfare*. London; 2005.

7. Cox BD, University of Cambridge Department of Community Medicine: **Health and. Lifestyle Survey : Seven Year Follow-up, 1991-1992 (HALS2) [computer file]** Colchester, Essex: The Data Archive [distributor]; 1995.

8. **Census** [http://www.gro-scotland.gov.uk/census/index.html]

9. **British Household Panel Survey** [http://www.iser.essex.ac.uk/survey/bhps]

10. Bajekal M, Primatesta P, Prior G: **Health Survey for England 2001**. London; 2003.

11. The Scottish Government: **The Scottish Health Survey - 2003 Results**. Edinburgh; 2005.

12. Office for National Statistics SaVSD: **Living in Britain: results from the 2003 General Household Survey.** London; 2004.

13. **Standard Occupational Classification** [http://www.ons.gov.uk/about-statistics/classifications/current/SOC2000/]

14. Petticrew M, Kearns A, Mason P, Hoy C: **The SHARP study: a quantitative and qualitative evaluation of the short-term outcomes of housing and neighbourhood renewal**. *BMC Public Health* 2009, **9**:415.

15. Stafford M, Nazroo J, Popay JM: **Tackling inequalities in health: evaluating the New Deal for Communities initiative**. *J Epidemiol Community Health* 2008, **62**:298–304.

16. Benzeval M, Der G, Ellaway A, Hunt K, Sweeting H, West P, Macintyre S: **Cohort Profile: West of Scotland 20-07 study: health in the community.** *Int J Epidemiol* 2009(38):1215-1223.

17. Macintyre S, Annandale E, Ecob R, Ford G, Hunt K, Jamieson B, MacIver S, West P, Wyke S: **The West of Scotland Twenty-07 Study: health in the community**. In *Readings for a new public health*. Edited by Martin C, McQueen D. Edinburgh: Edinburgh University Press; 1989:56-74.

18. **Quality Metric** [http://www.qualitymetric.com/WhatWeDo/GenericHealthSurveys/tabid/184/Default.aspx]
